# Effects of low and high levels of maternal nutrition consumed for the entirety of gestation on the development of muscle, adipose tissue, bone, and the organs of Wagyu cattle fetuses

**DOI:** 10.1111/asj.13600

**Published:** 2021-07-29

**Authors:** Yi Zhang, Kounosuke Otomaru, Kazunaga Oshima, Yuji Goto, Ichiro Oshima, Susumu Muroya, Mitsue Sano, Rena Saneshima, Yukiko Nagao, Aoi Kinoshita, Yasuko Okamura, Sanggun Roh, Akira Ohtsuka, Takafumi Gotoh

**Affiliations:** ^1^ Faculty of Agriculture Kagoshima University Kagoshima Japan; ^2^ Kuju Agricultural Research Center Kyushu University Taketa Oita Japan; ^3^ Joint Faculty of Veterinary Medicine Kagoshima University Kagoshima Japan; ^4^ Western Region Agricultural Research Center National Agriculture and Food Research Organization (NARO) Oda Shimane Japan; ^5^ Institute of Livestock and Grassland Science NARO Tsukuba Ibaraki Japan; ^6^ School of Human Cultures The University of Shiga prefecture Hikone Shiga Japan; ^7^ Graduate School of Agricultural Science Tohoku University Sendai Miyagi Japan

**Keywords:** fetal adipose tissue, fetal organ development, fetal skeletal muscle, maternal nutrition, Wagyu fetus

## Abstract

This study aimed to investigate the effects of high and low levels of energy intake during the entire gestation period on the skeletal muscle development, organ development, and adipose tissue accumulation in fetuses of Wagyu (Japanese Black) cows, a breed with highly marbled beef. Cows were allocated to a high‐nutrition (*n* = 6) group (fed 120% of the nutritional requirement) or low‐nutrition (*n* = 6) group (fed 60% of the nutritional requirement). The cows were artificially inseminated with semen from the same sire, and the fetuses were removed by cesarean section at 260 ± 8.3 days of fetal age and slaughtered. The whole‐body, total muscle, adipose, and bone masses of the fetal half‐carcasses were significantly higher in the high‐nutrition group than the low‐nutrition group (*p* = 0.0018, 0.009, 0.0004, and 0.0362, respectively). Fifteen of 20 individual muscles, five of six fat depots, nine of 17 organs, and seven of 12 bones that were investigated had significantly higher masses in the high‐nutrition group than the low‐nutrition group. The crude components and amino acid composition of the longissimus muscle significantly differed between the low‐ and high‐nutrition groups. These data indicate that maternal nutrition during gestation has a marked effect on the muscle, bone, and adipose tissue development of Wagyu cattle fetuses.

## 1 INTRODUCTION

In the past decade, it has been reported that the level of nutrition, and/or the dietary composition, and/or the timing of dietary changes in the dam during pregnancy can influence fetal organ development in livestock, via the placenta (Funston et al., 2010; Long et al., 2009; Long, Prado‐Cooper, et al., 2010; Meyer et al., 2010; Paradis et al., 2017; Underwood et al., 2010). In particular, effects on myogenesis, adipogenesis, fibrogenesis, and osteogenesis have been recorded. These developmental effects also significantly contribute to postnatal growth, maturation, and the resultant meat quality, which is considered crucial in the beef industry (Du & Zhu, 2009).

Du et al. (2005) measured the body mass (BM) and carcass mass of cattle fetuses at day 125 of their intrauterine development when 70% or 100% of the nutritional requirements of the dams had been met between days 30 and 125 of gestation and found no significant differences between the groups. Furthermore, Quigley et al. (2005) assessed the BM and muscle development of 75‐day‐old fetuses of sheep that had received 50% or 150% of their nutritional requirements for 24 days around periconception and found no differences in the body dimensions, organ development, or muscle masses. However, the group that received only 50% of its nutritional requirement had ~20% fewer myofibers than the group that received 150% of its nutritional requirement and had a lower secondary‐to‐primary fiber ratio, implying that secondary myofiber formation had occurred more slowly in the low‐nutrition group. Additionally, the protein: DNA ratio of the muscles of the low‐nutrition group tended to be lower. Therefore, the authors concluded that a restriction of feed intake during the periconception period reduces or delays myogenesis in fetal sheep. In contrast, Paradis et al. (2017) compared the effects of maternal nutrition at 100% or 85% of the required metabolic energy between 147 and 247 days post‐conception (mid‐to‐late pregnancy) on fetal development in crossbred cattle and found no significant differences in the whole‐body, heart, liver, kidney, or placental masses. However, differences in muscle DNA methylation and gene expression were observed. Thus, a relatively small restriction in the supply of metabolic energy to dams may not have immediate effects on phenotype, but does induce changes at the molecular level in their fetuses.

Fetal muscle development, which positively correlates with growth and meat production during the postnatal period, has been reported to be sensitive to the level of and timing of changes in maternal nutrition (Funston et al., 2010). Muscle mass is determined by myofiber number and type, which are fixed by the second trimester (Oksbjerg et al., 2004), as well as size. The hypertrophy of myofibers occurs from the third trimester until after birth (Long, Prado‐Cooper, et al., 2010). With respect to adipose tissue development, most adipocytes are generated during the fetal and early postnatal stages, and adipocyte hyperplasia is largely completed by weaning (Du, Yan, et al., 2010). Intramuscular fat accumulation (marbling) is thought to occur between the late fetal stage and ~250 days of age in beef cattle (Du et al., 2013a, 2013b). However, the mechanism of intramuscular adipose tissue formation remains unclear because few studies have been performed.

The distribution of nutrients from the placenta to the fetal organs is uneven. During the first trimester, vital organs, such as the brain, heart, and liver, are prioritized over skeletal muscle and adipose tissue (Bauman et al., 1982; Long et al., 2009; Meyer et al., 2010; Zhu et al., 2006). Fetal developmental programming has been poorly characterized to date because of its complexity. However, many studies have provided evidence that maternal nutrition has impacts on muscle and adipose development, not only in rodents but also in meat‐producing animals, in which carcass characteristics are important (Du et al., 2013a, 2013b; Lukaszewski et al., 2013). However, the relationship between maternal nutrition and carcass quality has not been well characterized. Many studies have assessed the carcass quality of the offspring of dams that had been exposed to differing levels of nutrition over various periods of gestation (Long et al., 2009, 2012; Long, Nijland, et al., 2010; Long, Prado‐Cooper, et al., 2010; Robinson et al., 2013). An Australian study showed relatively minor differences in meat characteristics (Greenwood et al., 2006), but equivalent studies have not been performed in Wagyu cattle.

Wagyu (Japanese Black) cattle produce excellent marbled beef because of a high level of intramuscular fat deposition (Gotoh et al., 2009). However, the influence of maternal nutrition on the fetal development and beef productivity of this breed has not been investigated. Therefore, we aimed to determine the effects of maternal nutrition on the fetal development, including the morphological phenotype and muscle gene expression, of Wagyu cattle. An understanding of the phenotypic responses to differences in maternal nutrition is a prerequisite for subsequent analysis of the underlying mechanisms and is of practical significance. To this end, in the present study, we compared the effects of high and low levels of energy intake during the entire gestation period on the fetal phenotype, including that of skeletal muscle, adipose tissue, bone, and other organs.

## 2 MATERIALS AND METHODS

This study was conducted at Kagoshima University and all the animal procedures were approved by the Kagoshima University Animal Care and Use Committee (A18007).

### 2.1 Animals, diets, and experimental design

A total of 32 Wagyu cows were prepared for this experiment. Thirty‐two multiparous Japanese Black cows were obtained from the Iriki farm (*n* = 12), Kagoshima University, and the Western Region Agricultural Research Center, NARO (*n* = 20), and randomly assigned to two diet groups according to BM: a low‐nutrition group and a high‐nutrition group. These cows were fed diets that were formulated to meet either 60% or 120% of their predicted requirements, based on the Japan Feeding Standard for Beef Cattle (JFSBC; NARO, 2008), from before they became pregnant. Using the standard diet model for pregnant Wagyu cows, which is based on the BM of the individual cows during their previous pregnancy, we formulated individual diets to meet 60% or 120% of their energy requirements using formula feed, total mixed ration (TMR) and rice straw (Table 1). Each animal was fed individually by using stanchions to lock each animal in until they entirely consumed each feed in the morning (09:00) and the afternoon (16:00). Cows were kept in drylots.

**TABLE 1:** Composition of the diets fed to the Wagyu cows for the duration of their gestation period

Item	Amount
Ingredient, % of diet (fresh matter basis)	
Formula feed^a^	7.30
Rice straw	31.61
Rice whole crop silage^b^	21.02
Dried timothy grass	7.88
Rye straw	7.55
Brown rice	3.94
Beer lees	3.28
Sugar cane pelltets	3.28
Tofu lees	2.63
Soy sauce cake	2.30
Sugar cane bagasse	1.97
Rice bran	1.31
Corn steep liquor	0.99
Condensed potetos distillers soluble	0.66
Reice trienol	0.66
Calcium	0.33
Water	3.28
Nutrient, % dry matter (DM) basis except DM itself	
Dry matter (DM)	68.00
Neutral detergent fiber (NDF)	56.10
Acid detergent fiber (ADF)	36.00
Ash	11.10
Crude protein (CP)	8.00
Calcium (Ca)	0.60
Phosphorus (P)	0.30
Predicted energy	
Metabolizable energy (ME), MJ/kg DM	8.56

^a^ Nisshin Marubeni Feed Co., Ltd. (Kagoshima, Japan). This formula feed (dry matter basis) contained 15.0% crude protein, 2.0% crude fat, and 10.0% ash; and yielded 70% Total Digestible Nutrients (TDN).

^b^ Kamichiku Group Co., Ltd. (Kagoshima, Japan). This rice whole‐crop silage was produced by Tachisuzuka (forage rice), and consisted of 6.54% crude protein, 48.0% NDF, and 26.2% NFC (dry matter basis).

All cows were synchronized using a controlled internal drug release (CIDR) device (Easybreed, InterAg Co. Ltd., Hamilton, New Zealand). All cows were inseminated with frozen male‐sorted semen from the same sire (Yurikatsuyasu, Kedaka line). We could finally obtain pregnant six cows from each group (low nutrition group: *n* = 6, high nutrition group: *n* = 6).

The total mixed ration (TMR) consisted of whole‐crop silage, composed of rice plant, dried timothy grass, rye straw, brown rice, beer lees, sugar cane pellets, tofu lees, soy sauce cake, sugar cane bagasse, rice bran, corn steep liquor, condensed sweet potato distillers' solubles, rice trienol, calcium, and water (Table 1). The final crude nutrient composition of the mixed feed, on a dry‐matter basis, was 56.1% neutral detergent fiber (NDF), 36.0% acid detergent fiber (ADF), 11.1% ash, 8.00% crude protein, 0.60% calcium, and 0.30% phosphorus (Table 1). The metabolizable energy (ME) provided by this feed was 8.56 MJ/kg dry matter (Table 1).

### 2.2 Slaughter and sample collection

Maternal BM was measured every month from the start of the study until the cows were transported to Kagoshima University Veterinary Teaching Hospital on day 260 ± 8.3 of gestation. A total of 12 fetuses were obtained by cesarean section and slaughtered: six from cows in the low‐nutrition group and six from the high‐nutrition group. The fetal BM, body length, and the masses of the organs (brain, hypophysis, thyroid, liver, kidneys, thymus, pancreas, spleen, heart, lungs, and testis) and fat depots were recorded. The gastrointestinal tract was divided into the esophagus, rumen, reticulum, omasum, and abomasum, rinsed with warm water, drained, cleaned, and individually weighed. The small intestine and large intestine were separated from the mesentery and from each other, and their masses were recorded after their contents had been removed. We collected adipose tissue samples from six depots: visceral fat from around the digestive organs, thoracic cavity fat, peritoneal cavity fat, perirenal fat, subcutaneous fat, and intermuscular fat from the right side of the carcass. We also carefully collected fat from the organs, muscles, and bones, and muscles from the right side of the carcass. The masses of 20 muscles were recorded: (1) *Mm. colli et dorsi* (*M. spinalis et semispinalis thoracis et cervices*, *semispinalis capitis*, and *longissimus thoracis*); (2) *Mm. congluli membri thoracici* (*M. trapezius*, *serratus ventralis thoracis*, *serratus ventralis cervicis*, *latissimus dorsi*, *and transversus thoracis*); (3) *Mm. membri thoracici* (*M. supraspinatus*, *infraspinatus*, *and triceps brachii*); and (4) *Mm. membri pelvini* (*M. psoas major*, *tensor fasciae latae*, *gluteus medius*, *vastus lateralis*, *rectus femoris*, *adductor*, *semimembranosus*, *and semitendinosus*).

After removing the fat, muscle, and connective tissue from the bones, we collected bone samples and measured the masses of the cervical, thoracic, lumbar, sacral (including the pelvis), and coccygeal vertebrae of the fetuses. We also measured the masses of 13 ribs, scapula, the carpi, radius, femur, and tibia from the right sides of the carcasses. Finally, we calculated the equivalent masses for each half‐carcass for the vertebrae, and the thoracic cavity fat, peritoneal cavity fat, and perirenal fat because we dissected the fetuses by hand and could not cut the spine in half, as occurs in slaughterhouses.

### 2.3 Measurement of the crude components, imidazole peptide, and amino acid composition of the longissimus muscle

We measured the moisture, crude protein, lipid, and crude ash contents of the longissimus muscles of the fetuses. We could not measure all samples because the volume of some samples that we used was limited (the measurement of the crude‐components, low nutrition group *n* = 5, high nutrition group *n* = 5; the measurement of imidazole peptide, and amino acid composition, low nutrition group *n* = 5, high nutrition group *n* = 3). The moisture and crude ash contents were measured using the AOAC method (2003), and the crude protein content was determined using hippuric acid as a standard and the nitrogen content, determined using the dry combustion method with an NC analyzer (JM1000; J‐Science Labs Inc., Kyoto, Japan), multiplied by a factor of 6.25. The extraction and quantification of lipids were performed according to Bligh and Dyer's (1959) method. For the quantification of proteinogenic amino acids, the muscle tissue was homogenized in 2% HClO4 and centrifuged to obtain the precipitated fraction, which was hydrolyzed in 6N HCl and the resultant hydrolysate used for the analysis. In addition, the concentration of free amino acids, carnosine, and anserine in the muscle tissue was measured. A liquid chromatography system with automated precolumn derivatization functionality was used in the analysis (Nexera X2; Shimadzu Corporation, Kyoto, Japan) as previously described by Azuma et al. (2016).

### 2.4 Statistical analyses

Maternal BM differences were analyzed using repeated measures ANOVA in EZR (Saitama Medical Center, Jichi Medical University, Saitama, Japan), which is a graphical user interface for R (R Foundation for Statistical Computing, Vienna, Austria). More precisely, it is a modified version of R commander, which was designed to add statistical functions that are frequently used in biostatistics (Kanda, 2013).

The BM and length of the fetus, the fetal carcass mass; and individual skeletal muscle, adipose tissue depot, and bone masses were analyzed using the GLM procedure of SAS (SAS University Edition; SAS Inst. Inc. 2014, Cary, NC, USA). The masses of the fetal organs at slaughter were analyzed using the GLM procedure of SAS, with diet in the model statement. The results are reported at least squares means ± standard error (SE), and effects were considered to be significant at *p* ≤ 0.05 and to represent a trend at *p* ≤ 0.10.

## 3 RESULTS

### 3.1 Differences in the body masses of the dams between the low‐ and high‐nutrition groups

**FIGURE 1:**
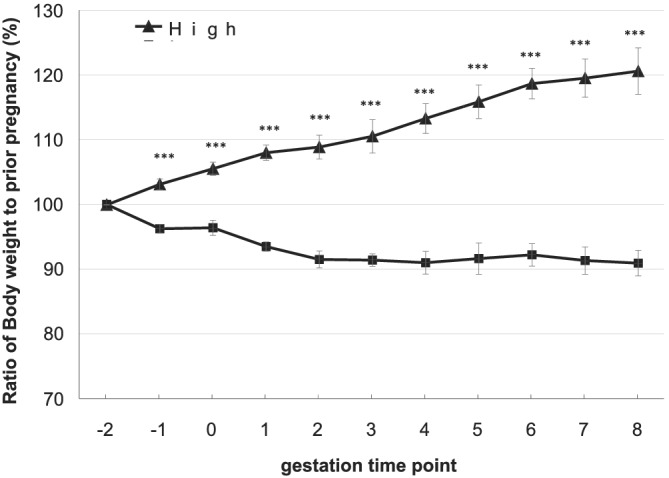
Change in the body mass of dams in the low and high nutrition groups between 2 months prior to fertilization and 8 months of gestation. Data are mean ± standard error. ***p < 0.001 between the low and high nutrition groups at that time point

Regarding the nutritional treatment in this study, there were no feed residues during this experiment in low‐ and high‐nutrition groups. The BM of dams at the initiation of the test diets was similar for dams in the low‐ and the high‐nutrition groups (497 ± 15 kg and 491 ± 8 kg, respectively; *p* = 0.751) (Figure 1). However, the BM of the dams was significantly different between the two groups from 1 month prior to fertilization (*p* < 0.001; Table 1). The cows in the low‐nutrition group lost weight, such that their BM was 90% of the starting mass at 8 months of gestation. In contrast, the cows in the high‐nutrition group gained weight, such that their BM was 120% of their starting mass at the same time point (Figure 1).

### 3.2 Comparisons of the general muscle, fat, and bone masses of fetuses in the low‐and high‐nutrition groups

The BM, half‐carcass mass (right side), and half‐carcass composition (muscle, fat, and bone) were influenced by maternal nutrition level. The BM of the fetuses at 260 ± 8.3 days (around 8.5 months) of gestation was lower in the low‐nutrition group than in the high‐nutrition group (23,390 ± 2,156 g and 32,650 ± 449 g, respectively; *p* = 0.0018, Table 2). Fetal body length (from the level of the scapula position to that of the ischium) did not differ between the high‐ and low‐nutrition groups (*p* = 0.946). Similarly, the muscle, fat, and bone masses were lower in the low‐nutrition group than in the high‐nutrition group (*p* = 0.0090, 0.0004, and 0.0362, respectively; Table 2).

**TABLE 2:** Effects of a low or high nutritional status of Wagyu cows during the entirety of gestation on the fetal body length, body mass, carcass mass, and half carcass masses of muscle, fat, and bone

Item	Weight (g)				Ratio^a^ (%)		
	Low^b^	High^b^	*P*‐value	High/low^c^	Low	High	*p* value
Animals, no.	6	6			6	6	
Fetal body length, cm	54.3 ± 2.9	60.3 ± 1.4	0.9460				
Fetal BW, g	23390.0 ± 2155.8	32653.0 ± 448.5	0.0018	1.396			
Right half carcas weight, g	5720.0 ± 544.0	8033.0 ± 71.0	0.0067	1.404			
Muscle weight^d^, g	3497.0 ± 366.3	4972.5 ± 139.5	0.0090	1.422	61.2 ± 1.0	62.0 ± 1	0.5660
Fat weight^d^, g	333.0 ± 19.0	708.0 ± 51.3	0.0004	2.126	5.4 ± 0.7	8.1 ± 1	0.0230
Bone weight^d^, g	1890.0 ± 177.2	2353.0 ± 54.0	0.0362	1.245	33.4 ± 0.4	29.8 ± 1	0.0042

*Note*: Data are mean ± standard error.

^a^ Ratio of mass relative to the calculated fetal half‐carcass (right side) mass.

^b^ Wagyu cows were fed diets providing a low (60%) or high nutrition (120%) level, according to the JFSBC (NARO, 2008) nutritional requirements.

^c^ Fold difference between the high‐nutrition group and the low‐nutrition group for items with *p* < 0.10.

^d^ Mass calculated for the right half of the carcass.

The ratios of the muscle, fat, and bone masses to the calculated half‐carcass (right side) masses were influenced by maternal nutrition level (Table 2). The percentage fat by mass for the half‐carcass was lower in fetuses of the low‐nutrition group than in the high‐nutrition group (*p* = 0.0230), whereas the percentage bone by mass was higher in fetuses of the low‐nutrition group than in the high‐nutrition group (*p* = 0.0042). However, no difference in the percentage muscle by mass was found between the low‐ and high‐nutrition groups.

### 3.3 Differences in individual muscle, fat depot, and bone development between fetuses of the low‐ and high‐nutrition groups

The masses of 15 muscles out of 20 muscles we investigated were lower in fetuses of the low‐nutrition group (all *p* < 0.05; Table 3). The exceptions were the spinalis et semispinalis thoracis et cervices, transversus thoracis, and semimembranosus muscles. Furthermore, the masses of the infraspinatus, biceps femoris, and tensor fasciae latae tended to be lower in fetuses of the low‐nutrition group than in those of the high‐nutrition group (*p* = 0.058, 0.075, and 0.050, respectively). With respect to the ratios of individual muscle masses to that of the half‐carcass, only the adductor muscle had a lower percentage mass in fetuses of the low‐nutrition group, compared to the high‐nutrition group (*p* = 0.0260).

**TABLE 3:** Effects of low or high nutritional status of Wagyu cows during the entirety of gestation on the muscle, fat, and bone masses and their ratios relative to the half‐carcass mass of the fetuses

Item	Weight^a^ (g)				Ratio^b^ (%)		
	Low^c^	High^c^	*p* value	High/low^d^	Low	High	*p* value
No. of animal	6	6			6	6	
Muscle							
*Mm. colli et dorsi*							
*Spinalis et semispinalis thoracis et cervices*	74.2 ± 8.3	90.2 ± 6.0	0.166				
*Semispinalis capitis*	80.7 ± 8.8	110.8 ± 4.9	0.037	1.373	1.4	1.4	0.621
*Longissimus thoracis*	187.0 ± 22.4	237.2 ± 6.1	0.010	1.268	3.3	3.4	0.337
*Mm. cinguli membri thoracici*							
*Trapezius*	48.3 ± 6.3	70.2 ± 4.7	0.028	1.453	0.8	0.9	0.592
*Serratus ventralis thoracis*	36.2 ± 4.1	51.5 ± 2.4	0.014	1.423	0.6	0.6	0.796
*Serratus ventralis cervicis*	82.7 ± 9.6	119.2 ± 4.7	0.015	1.441	1.5	1.5	0.454
*Latissimus dorsi*	67.5 ± 7.6	103.7 ± 3.0	0.005	1.536	1.2	1.3	0.165
*Transversus thoracis*	58.3 ± 7.0	69.8 ± 3.8	0.168				
*Mm. membri thoracici*							
*Supraspinatus*	56.7 ± 4.7	83.7 ± 3.6	0.001	1.476	1.0	1.0	0.392
*Infraspinatus*	78.9 ± 9.6	114.2 ± 10.3	0.058	1.447	1.4	1.4	0.694
*Triceps brachii*	113.8 ± 13.2	162.3 ± 6.1	0.014	1.426	2.0	2.0	0.642
*Mm. membri pelvini*							
*Biceps femoris*	212.7 ± 26.8	273.3 ± 9.4	0.075	1.285	3.7	3.4	0.175
*Psoas major*	60.8 ± 7.2	79.5 ± 5.0	0.039	1.308	1.1	1.0	0.539
*Tensor fasciae latae*	33.7 ± 4.3	44.5 ± 1.0	0.050	1.320	0.6	0.6	0.386
*Gluteus medius*	90.3 ± 10.9	131.8 ± 2.8	0.013	1.460	1.6	1.6	0.360
*Vastus lateralis*	85.5 ± 11.2	126.0 ± 5.2	0.014	1.474	1.5	1.6	0.401
*Rectus femoris*	79.3 ± 8.7	112.3 ± 2.5	0.014	1.416	1.4	1.4	0.699
*Adductor*	53.0 ± 4.1	91.2 ± 5.8	0.012	1.721	1.0	1.1	0.026
*Semimembranosus*	158.3 ± 33.7	201.2 ± 5.6	0.152				
*Semitendinosus*	64.5 ± 8.0	91.0 ± 1.5	0.015	1.411	1.1	1.1	0.791
Others muscles	1774.5 ± 366.3	2573.0 ± 89.6	0.004	1.450	31.5	32.0	0.361
Fat							
Thoracic cavity^e^	25.5 ± 2.4	37.6 ± 3.5	0.018	1.475	0.5	0.5	0.937
Peritoneal cavity^e^	66.3 ± 6.8	114.9 ± 12.5	0.007	1.733	1.2	1.4	0.240
Subcutaneous	41.5 ± 5.3	125.8 ± 19.2	0.006	3.032	0.8	1.6	0.032
Intermuscular	159.0 ± 11.2	369.8 ± 33.5	0.001	2.326	3.0	4.6	0.026
						Ratio to BW	
Perirenal^f^ (total)	82.2 ± 8.3	119.2 ± 6.7	0.006	1.450	0.4	0.4	0.769
Visceral^f^	84.4 ± 14.6	128.8 ± 16.6	0.060	1.526	0.4	0.4	0.555
Bone							
*Sternum*	77.0 ± 8.5	91.7 ± 4.3	0.164				
*Vertebrae Cervicales*	350.0 ± 30.5	441.7 ± 15.5	0.030	1.262	3.1	2.7	0.045
*Vertebrae Thoracalis*	402.3 ± 34.6	510.0 ± 14.2	0.025	1.268	3.6	3.1	0.012
*Vertebrae Lumbales*	209.5 ± 25.9	257.5 ± 9.8	0.130				
*Vertebrae Sacrales (including pelvis)*	366.7 ± 30.0	470.8 ± 15.8	0.016	1.284	3.3	2.9	0.031
*Vertebrae Coccygeal*	32.3 ± 4.0	42.0 ± 1.0	0.059	1.300			
*Ribs*	232.0 ± 23.2	286.8 ± 8.0	0.065	1.236	4.1	3.5	0.001
*Scapula*	81.7 ± 7.4	111.0 ± 2.4	0.009	1.359	1.4	1.4	0.198
*Ossa carpi*	188.7 ± 17.1	250.5 ± 10.3	0.014	1.328	3.3	3.1	0.229
*Radius*	177.8 ± 17.6	230.7 ± 11.8	0.035	1.297	3.1	2.8	0.190
*Os femoris*	272.3 ± 26.3	357.8 ± 11.0	0.021	1.314	4.8	4.4	0.178
*Tibia*	235.7 ± 39.1	268.0 ± 7.8	0.452				

*Note*: Data are mean ± standard error.

^a^ Mass, expressed in grams.

^b^ Ratio relative to the calculated right‐side carcass mass of the fetus.

^c^ Fold difference between the high‐nutrition group and the low‐nutrition group for items with *p* < 0.10.

^d^ The adipose tissue surrounding the kidney or intestine.

^e^ The weights of adipose tissue of the thoracic or peritoneal cavities of the right side of the carcass.

^f^ Wagyu cows were fed diets that provided low (60%) or high (120%) levels of nutrition, according to the JFSBC (NARO, 2008) nutritional requirements.

The masses of most of the fat depots (thoracic cavity, peritoneal cavity, subcutaneous, intermuscular, and perirenal fat) were influenced by maternal nutrition level (Table 3). The masses of the thoracic cavity, peritoneal cavity, subcutaneous, intermuscular, and perirenal fat depots were lower in the low‐nutrition group than in the high‐nutrition group (*p* = 0.020, 0.010, 0.006, 0.001, and 0.006, respectively; Table 3). Additionally, the visceral fat mass tended to be lower in the low‐nutrition group (*p* = 0.060). With respect to the ratios of the masses of the fat depots to that of the half‐carcass, the subcutaneous and intermuscular fat percentages were lower in the low‐nutrition group (*p* = 0.0319 and 0.0261, respectively; Table 3). However, the ratios with respect to the perirenal and visceral fat depots did not differ between the two groups (*p* = 0.7685 and 0.552, respectively; Table 3).

The masses of a number of bones/groups of bones [cervical vertebrae, thoracic vertebrae, sacral vertebrae (including pelvis), scapula, carpi, radius, and femur] were lower in the low‐nutrition group (*p* = 0.030, 0.025, 0.016, 0.009, 0.014, 0.035, and 0.021, respectively; Table 3). Furthermore, the masses of the coccygeal vertebrae and ribs tended to be lower in the low‐nutrition group (*p* = 0.059 and 0.065, respectively). However, the masses of the sternum, lumbar vertebrae, and tibia did not differ between the low‐ and the high‐nutrition groups (Table 3).

With respect to the ratios of the masses of individual bones/bone groups to that of the half‐carcass, those for the sternum, cervical vertebrae, thoracic vertebrae, sacral vertebrae (including the pelvis), and ribs were higher in the low‐nutrition group than in the high‐nutrition group (*p* = 0.0329, 0.0446, 0.0120, 0.0313, and 0.0014, respectively, Table 3). Additionally, the ratios for the lumbar vertebrae and tibia tended to be higher in the low‐nutrition group (*p* = 0.0669 and 0.0654, respectively).

### 3.4 Differences in the organ development of fetuses in the low‐ and high‐nutrition groups

The development of many organs was influenced by maternal nutrition level (Table 4). The masses of the liver (*p* = 0.018), kidney (right; *p* = 0.033), thymus (*p* = 0.009), spleen (*p* = 0.012), heart (*p* = 0.026), lung (*p* = 0.005), rumen (*p* = 0.038), omasum (*p* = 0.028), and large intestine (*p* = 0.019) were lower in fetuses of the low‐nutrition group than in those of the high‐nutrition group. Furthermore, the mass of the brain tended to be lower in the low‐nutrition group (*p* = 0.0730). However, no significant differences in the masses of the other organs were found (Table 4).

**TABLE 4:** Effects of a low or high nutritional status in Wagyu cows for the entirety of their gestation on the mass and mass ratios for the fetal organs

Item	Weight^a^ (g)				Ratio^b^		
	Low^c^	High^c^	*p* value	High/low^d^	Low	High	*p* value
No. of animal	6	6			6	6	
Brain	186.4 ± 9.2	210.0 ± 7.2	0.073	1.127	8.3 ± 0.7	6.4 ± 0.3	0.039
Hypophysis	0.3 ± 0.1	0.5 ± 0.1	0.114				
Thyroid	12.4 ± 3.0	10.2 ± 1.0	0.509				
Liver	487.3 ± 41.8	627.5 ± 16.2	0.018	1.287	21.0 ± 0.7	19.0 ± 0.4	0.044
Kidney (right)	43.3 ± 4.3	55.8 ± 1.8	0.033	1.289	1.9 ± 0.1	1.7 ± 0.1	0.140
Thymus	118.3 ± 19.7	212.0 ± 21.2	0.009	1.793	5.0 ± 0.7	6.4 ± 0.7	0.166
Pancreas	11.9 ± 1.2	12.7 ± 1.6	0.666		0.5 ± 0.0	0.4 ± 0.1	0.069
Spleen	50.9 ± 6.6	76.3 ± 5.0	0.012	1.499	2.1 ± 0.1	2.3 ± 0.2	0.341
Heart	173.7 ± 14.8	220.0 ± 7.7	0.026	1.267	7.5 ± 0.4	6.7 ± 0.2	0.090
Lung	508.3 ± 45.0	730.7 ± 42.8	0.005	1.437	21.8 ± 0.6	22.2 ± 1.3	0.812
Rumen	73.7 ± 8.2	99.3 ± 6.9	0.038	1.348	3.1 ± 0.1	3.0 ± 0.2	0.629
Reticulum	15.8 ± 2.3	20.4 ± 1.7	0.135				
Omasum	32.7 ± 3.3	42.5 ± 1.5	0.028	1.302	1.4 ± 0.1	1.3 ± 0.0	0.146
Abomasum	92.7 ± 11.9	122.5 ± 11.3	0.100				
Testis	2.2 ± 0.7	3.2 ± 0.5	0.280				
Small intestine	280.9 ± 18.2	323.2 ± 17.2	0.126				
Large intestine	61.6 ± 8.7	93.1 ± 7.0	0.019	1.511	2.6 ± 0.2	2.8 ± 0.2	0.551

*Note*: Data are mean ± standard error.

^a^ Mass, expressed in grams.

^b^ Ratio, expressed as gram of organ per kilogram of fetal body mass.

^c^ Wagyu cows were fed diets that provided low (60%) or high (120%) levels of nutrition, according to the JFSBC (NARO, 2008) nutritional requirements.

^d^ Fold difference between the high‐nutrition group and the low‐nutrition group for items with *p* < 0.10.

With respect to the mass ratios (gram of organ per kilogram of fetal BM), only that of the brain was significantly higher in the low‐nutrition group than in the high‐nutrition group (*p* = 0.039), but those of the pancreas and heart tended to be higher (*p* = 0.069 and 0.090, respectively). No significant differences in the mass ratios for any of the other organs were observed (Table 4).

### 3.5 Differences in the crude components, amino acid composition, and imidazole dipeptide content of longissimus muscle in fetuses of the low‐ and high‐nutrition groups

The crude components of the longissimus muscles of the fetuses are shown in Table 5. Only the percentage crude ash was lower in the low‐nutrition group than in the high‐nutrition group (*p* = 0.035); no differences with respect to moisture, crude protein, or crude fat were found between the groups (*p* = 0.434, 0.902, and 0.418, respectively).

**TABLE 5:** Crude components of the longissimus muscle of Wagyu fetuses in the maternal low‐ and high‐nutrition groups

Items	Low^a^ (%)	High^a^ (%)	*p* value
No. of animal	5	5	
Moisture	77.90 ± 0.25	78.70 ± 1.00	0.434
Crude protein	19.25 ± 1.00	19.50 ± 1.80	0.902
Crude fat (ether extract)	1.78 ± 0.40	2.20 ± 0.55	0.418
Crude ash	1.06 ± 0.01	1.10 ± 0.01	0.035

*Note*: Data are mean ± standard error.

^a^ Wagyu cows were fed diets that provided low (60%) or high (120%) levels of nutrition, according to the JFSBC (NARO, 2008) nutritional requirements.

Analysis of the proteinogenic amino acids showed that only the methionine content of the longissimus muscle was lower in fetuses of the low‐nutrition group than in those of the high‐nutrition group (*p* = 0.012, Table 5). However, the glycine content tended to be higher in fetuses of the low‐nutrition group (*p* = 0.099). Analysis of the imidazole dipeptides, such as carnosine and anserine, and free amino acids in the longissimus muscle (Table 5) showed that the contents of aspartic and glutamic acids were higher in the low‐nutrition group (*p* = 0.003 and 0.007, respectively). No differences in the concentrations of the other free amino acids in the longissimus muscle were identified (Table 6). Additionally, the contents of carnosine and anserine did not differ between the groups (Table 6).

**TABLE 6:** Proteinogenic amino acids, free amino acid, and imidazole dipeptide concentrations in the longissimus muscles of Wagyu fetuses in the maternal low‐ and high‐nutrition groups

item	proteinogenic amino acid			free amino acid and Imidazole peptide		
	Low^a^	High^a^	*p*‐value	Low^a^	High^a^	*p*‐value
No. of animal	5	3		5	3	
Alanine	1,300 ± 32	1,233 ± 56	0.303	36.8 ± 3.44	41.8 ± 2.89	0.179
Arginine	1,362 ± 27	1,317 ± 49	0.403	3.47 ± 0.63	3.60 ± 0.78	0.453
Aspartic acid	1824 ± 34	1933 ± 52	0.112	1.67 ± 0.13	2.86 ± 0.24	0.003
Glutamic acid	4,618 ± 127	4,740 ± 164	0.578	66.4 ± 4.05	85.1 ± 0.72	0.007
Glycine	1,346 ± 114	1,063 ± 84	0.099	19.5 ± 2.11	22.3 ± 2.63	0.217
Histidine	418 ± 28	433 ± 28	0.731	—	—	—
Isoleucine	908 ± 49	957 ± 35	0.511	0.81 ± 0.15	0.91 ± 0.12	0.335
Leucine	1832 ± 80	1880 ± 64	0.695	1.08 ± 0.20	1.07 ± 0.23	0.483
Lysine	1776 ± 133	1833 ± 139	0.788	—	—	—
Methionine	398 ± 10	453 ± 12	0.012	—	—	—
Phenylalanine	786 ± 27	797 ± 18	0.790	1.25 ± 0.15	1.03 ± 0.10	0.166
Proline	994 ± 55	853 ± 69	0.165	2.03 ± 0.44	2.03 ± 0.29	0.995
Serine	924 ± 41	903 ± 56	0.771	1.47 ± 0.84	1.03 ± 0.20	0.711
Threonine	816 ± 32	850 ± 38	0.526	0.86 ± 0.10	0.98 ± 0.17	0.273
Tyrosine	808 ± 40	857 ± 22	0.420	—	—	—
Valine	918 ± 32	940 ± 21	0.640	1.09 ± 0.13	1.43 ± 0.50	0.218
NH3	570 ± 102	437 ± 9	0.366	12.3 ± 0.89	14.0 ± 0.19	0.111
Carnosine	—	—	—	137.4 ± 15.18	118.5 ± 13.06	0.216
Anserine	—	—	—	8.0 ± 1.55	7.8 ± 1.76	0.460

*Note:* Data are mean ± standard error. Units: mg/100 g.

^a^ Wagyu cows were fed diets that provided low (60%) or high (120%) levels of nutrition, according to the JFSBC (NARO, 2008) nutritional requirements.

## 4 DISCUSSION

### 4.1 Effects of maternal nutrition on general muscle, fat and bone development

A high level of maternal nutrition has profound effects on offspring mass at birth, weaning, and slaughter, relative to a lower level (Greenwood et al., 2005; Greenwood & Café, 2007). It has also been reported that the time until the end of fattening in feedlot beef cattle is significantly shorter if a higher plane of maternal nutrition is achieved (Greenwood et al., 2005; Greenwood & Café, 2007). Furthermore, Du, Tong, et al. (2010) and Du et al. (2011, 2013a, 2013b, 2015) have shown effects of maternal nutrition on myogenesis, adipogenesis, and fibrogenesis in ruminant offspring. In the present study, we found that the muscle, fat, and bone masses of the fetal carcasses were significantly higher when the maternal level of nutrition was high for the entire gestation period. The fold‐difference in fat mass in the high‐ versus the low‐nutrition group was quite large (2.13‐fold) compared with those for the muscle (1.42‐fold) and bone (1.25‐fold). Furthermore, with respect to carcass composition, the mass ratio for the fat was higher and that for the bone was lower in the high‐nutrition group versus the low‐nutrition group. Thus, maternal nutrition level affects not only fetal muscle, bone, and organ development, but also fetal adipose tissue accumulation.

The carcass composition of conventionally fattened Wagyu cattle has been reported to be 47.4% (238 kg), 41.6% (208 kg), and 10.6% (53 kg), for muscle, fat, and bone, respectively (Gotoh et al., 2009). If the fetal data are used to estimate the fat percentage of the carcass after fattening, the carcass fat percentage of 5.4%–8.1% (666–1416 g) would be expected to change to 41.6% (208 kg; an 147‐ to 312‐fold increase) at the slaughter age of 26 months. In contrast, the percentages of muscle, fat, and bone in the carcasses of conventionally fattened German Angus have been reported to be 66.2% (237 kg), 22.3% (80 kg), and 11.5% (41 kg), respectively (Gotoh et al., 2009). Mao et al. (2008) reported that the body fat (including carcass fat, fat in organs, and fat in internal depots) of German Angus fetus at month 9 indicated 426.7 g. This body fat mass of German Angus fetus was clearly lower compared to just carcass fat mass of the low‐nutrition group of Wagyu fetus (at month 8.5; 666 g). On the other hand, the differences of final carcass fat mass between fattened German Angus and Wagyu indicated a 2.6‐fold change (80 and 208 kg, respectively; Gotoh et al., 2009). This thing would reveal marked differences in adipogenesis activity during the fetal stage between Wagyu and German Angus. However, further investigation is required to inquire into this fact.

Fetal growth in cattle can be divided into three phases. The first trimester is a period of hyperplasia, with little cellular hypertrophy; the second trimester is a transitional stage, during which there is a change in emphasis from hyperplasia to hypertrophy; and during the third trimester cellular hypertrophy predominates (Du & Zhu, 2009). In the present study, the test diets were consumed from at least 2 months prior to fertilization and the fetuses were studied at 8.5 months of gestation. Therefore, it was presumed that fetuses in the high‐nutrition group would have a larger number of larger cells and higher tissue masses than those in the low‐nutrition group. In contrast, it was predicted that muscle and adipose tissue development would be sacrificed in favor of more critical organs, such as the brain, heart, and liver. Indeed, fetal BM was higher in the high‐nutrition group, and this difference was contributed to by the muscle, bone, and fat at 8.5 months of gestation. Additionally, adipose tissue accumulation contributed to the total because excess nutrients would have been stored in this tissue.

### 4.2 Effects of maternal nutrition on individual muscle development

In the present study, the masses of many muscles were 1.2‐ to 1.7‐fold higher in the high‐nutrition group. Maternal nutrition programs fetal development, especially that of skeletal muscle. Myofiber formation (secondary myogenesis) predominantly occurs in the late phase of gestation; however, mid‐gestation is the most important time for skeletal muscle development (Du, Tong, et al., 2010; Greenwood et al., 2000). Several previous studies have shown that nutrient restriction during early and mid‐gestation reduces myofiber number in sheep (Quigley et al., 2005; Zhu et al., 2004), pigs (Dwyer et al., 1994; Zhu et al., 2006), and guinea pigs (Ward & Stickland, 1991). In contrast, a restriction of maternal nutrition during late gestation has a major impact on myofiber size, but not the number of muscle fibers (Du & Zhu, 2009; Greenwood et al., 1999).

In the present study, out of the 20 skeletal muscles that were weighed, fifteen were found to be significantly heavier in the high‐nutrition group than in the low‐nutrition group. Furthermore, another two (adductor, 1.72 times larger; and latissimus dorsi, 1.54 times larger) tended to be heavier (Table 3). The longissimus and the biceps femoris muscles, which are the largest skeletal muscles in Wagyu cattle (Gotoh et al., 1999), were ~1.3 times heavier in the high‐nutrition group than in the low‐nutrition group. The forelimb girdle muscles (cinguli thoracici) were ~1.4 times heavier and were well developed in the high‐nutrition group, but showed less development in the low‐nutrition group. The thoracic and thigh muscles (supraspinatus, infraspinatus, triceps brachii, gluteus medius, vastus lateralis, rectus femoris, and semitendinosus muscles) were also ~1.4 times heavier in the high‐nutrition group than in the low‐nutrition group, and were also relatively well developed. These muscles are the largest ones in adult Wagyu cattle; therefore, it is surprising that the longissimus and biceps femoris muscles were not better developed at this stage of gestation.

Previous studies conducted in sheep and cattle have shown that maternal nutrition during early‐to mid‐gestation affects the number of myofibers, rather than muscle mass (Paradis et al., 2017; Quigley et al., 2005). Gauvin et al. (2020) reported that maternal nutrition at 100%, 60%, and 140% of the recommended levels in sheep from day 30 of gestation to days 45, 90, or 135, or parturition, affected the ratio of secondary‐to‐primary muscle fibers in the longissimus muscle (*p* < 0.05), but not in the semitendinosus or triceps brachii, and caused differences in muscle gene expression. They concluded that poor maternal nutrition during gestation affects offspring muscle growth during early fetal development and that the effects persist throughout fetal development. These muscle type‐specific effects of maternal diet mean that it is important to evaluate more than one type of muscle to fully elucidate the effects of maternal diet on offspring muscle development.

### 4.3 Effects of maternal nutrition on adipose tissue development and adipogenesis specifically

In the present study, maternal nutrition markedly affected the adipose tissue development of the fetuses. Adipose tissue is mainly found in four sites in livestock: (1) visceral depots, (2) subcutaneous depots, (3) intermuscular depots, and (4) intramuscular depots. Wagyu cattle accumulate more intramuscular adipocytes than European cattle (Gotoh et al., 2009). Most adipocytes form during the fetal and early postnatal stages, and adipocyte hyperplasia is largely complete in perirenal fat by birth (Du, Yan, et al., 2010). However, Goessling et al. (2009) have suggested that the total number of adipocytes is not set until adolescence, and the visceral adipose depots form between mid‐gestation and the early postnatal period (Robelin, 1981).

The nutrients supplied to the mother are partitioned among the adipose tissue depots and the degree of fat accumulation varies according to the level of nutrition. In the present study, the masses of the thoracic cavity, perirenal, and visceral fat depots were 1.48, 1.45, and 1.53 times larger, respectively, in the high‐nutrition group than in the low‐nutrition group. Moreover, the mass of the peritoneal cavity fat was 1.73 times higher, that of the intermuscular fat was 2.33 times higher, and that of the subcutaneous fat was 3.03 times higher. This implies that excess nutrients tend to predominantly accumulate in the subcutaneous fat and intermuscular fat depots of these fetuses. It is generally stated that, during the postnatal period, fat accumulation occurs first in visceral adipose tissue, followed by subcutaneous adipose tissue, lastly in intramuscular adipose tissue (Sainz & Hasting, 2000). Although there was no significant difference in intramuscular fat content of longissimus muscle between the nutritional groups in this study, this statement is consistent with the trend of fat accumulation in our results.

### 4.4 Effects of maternal nutrition on bone development

In the present study, we also found effects of maternal nutrition on fetal bone development. A number of bones/bone groups, including the lumbar vertebrae and fibula, were significantly heavier in the high‐nutrition group than in the low‐nutrition group, although some, such as the sternum, were not. The ratios of these bone masses to BM were 1.2–1.3 times higher in the high‐nutrition group than in the low‐nutrition group. Thus, the effect of maternal nutrition on bone development was relatively small in comparison to its effects on skeletal muscle and adipose tissue development. High bone mass at skeletal maturity reduces the risks of osteoporosis and fracture (Eastell & Lambert, 2002), and in ruminants, this would be associated with superior animal health and meat production. Estêvāo et al. (2012) reported effects of maternal undernutrition in late gestation on bone and muscle development in sheep. Hindlimb muscle and bone samples were collected from offspring around 1 month of age. Bone length and mass were unaffected by nutrient deprivation in utero, but the calcification of the tibias was delayed postpartum. In the present study, we found significant differences in the bone development of the offspring of cows that had differing nutritional status during the whole of gestation. Therefore, further research is required to characterize the effects of nutrition on bone development in more detail.

### 4.5 Effects of maternal nutrition on organ development

During gestation, certain organs, namely the brain, heart, and liver, whose development is important for survival, are thought to preferentially receive nutrients (Zhu et al., 2006); this implies that skeletal muscle and adipose tissue have a lower priority. Thus, if nutrient supply is restricted, the development of muscle and adipose would be retarded. In the present study, no differences in the masses of the pituitary gland, thyroid gland, reticulum, abomasum, pancreas, testis, or small intestine were found between the two groups, which suggests that these organs are not affected by the levels of maternal nutrition that were tested. However, significant differences were identified between the two groups in the masses of other organs. Most of the organs were 1.13–1.35 times heavier in the high‐nutrition group than in the low‐nutrition group, but the spleen and large intestine were 1.50 and 1.51 times heavier, respectively. This may imply that the higher maternal nutritional plane is associated with an increase in hematopoiesis in the spleen and superior absorption of water and minerals in the large intestine. Furthermore, the thymus of fetuses in the high‐nutrition group was 1.79 times heavier than that in the low‐nutrition group. The larger mass of thymus at neonate in the high nutrition group might have more advantage against disease during newborn period because the thymus has important roles in the immune system, such as in T‐cell differentiation (Gasisova et al., 2016).

The four stomach chambers of ruminants originate early in development. Initially, the rumen is larger than the abomasum, but they are a similar size at 7 months of gestation (Becker et al., 1951; Masri et al., 2017), and immediately after birth the rumen is smaller than the abomasum. In the present study, a significant difference in rumen mass was observed between the groups, but not in abomasal mass. However, the rumen was lighter than the abomasum in both groups. After birth, the rumen develops further when the animal starts to eat a solid diet. Duarte et al. (2013) investigated the effects of maternal nutrition on the development of the gastrointestinal tract in feed‐restricted and ad libitum‐fed Nellore cows, and found no significant differences in the mass of the fetal gastrointestinal tract between the two groups. However, the lengths of the small intestine and its villi were larger in the feed‐restricted group than in the ad libitum‐fed group. This implies that the feed‐restricted calves would have larger absorptive surfaces in their small intestines. Although the length of the gastrointestinal tract was not measured in the present study, there was no difference in mass; poor nutrition may have had a similar effect in the Wagyu calves.

Paradis et al. (2017) determined how provision of 85% of the total metabolizable energy requirement to cows during mid‐to‐late (days 147–247) gestation influenced the phenotypic development of Angus/Simmental cross‐bred calves, compared with the provision of 140%. They found no differences in fetal BM; body length; chest circumference; or the heart, liver, kidney, or placental masses of fetuses in the two groups at 247 days of gestation. These findings suggest that maternal nutrition during mid‐to‐late gestation does not affect fetal phenotypic development. In contrast, in the present study, we found differing fetal, heart, liver, and kidney masses between high (120% of the required energy) and low (60% of the required energy) nutritional status throughout gestation. The differences in the levels of nutrition provided in each study may explain this disparity. Moreover, we found differences in the mRNA and microRNA expression and myofiber density in the muscles of the two groups (unpublished data). Early‐to‐mid gestation nutrition, which affects the early development of the embryo/fetus, is likely to facilitate the later phenotypic development of the fetus by affecting the supply of nutrients in late gestation.

### 4.6 Effects of maternal nutrition on the crude components, amino acid composition, and imidazole peptide content of the longissimus muscles of the fetus

We found that the level of maternal nutrition affected the crude components of the longissimus muscles of the fetuses. The ash content was significantly higher in the high‐nutrition group than in the low‐nutrition group, but there were no significant differences in the moisture, crude protein, or fat contents. Although the feed based on TMR used in this study contained 11.1% ash (DM basis; Table 1), further research is required to determine why there is a difference in mineral content of the muscle between the groups via maternal body and placenta.

As discussed above, there were significant differences in fat deposition between the groups, but there were no differences in the fat extract percentage or intramuscular fat content of the longissimus muscle. Several studies have identified the period between weaning and ~250 days of age as the “marbling window.” For example, Du et al. (2013a, 2013b) have suggested that the marbling process occurs from the late stage of gestation until ~250 days of age in beef cattle. However, the mechanism for the accumulation of intramuscular adipocytes remains unclear. Adipogenesis occurs principally from the mid‐to‐late fetal stage until ~250 days of age, but a difference in the fat content of the longissimus muscle could not be detected at 261 days of gestation in the present study. However, it is possible that preadipocytes with small or no fat droplets may already be present in the extracellular matrix at this time point. They arise through differentiation of adipocyte progenitor cells in skeletal muscles into adipocytes in fetuses that are provided with sufficient nutrition. These adipocytes may proliferate and contribute to the optimal fattening of the adult cattle. We plan to analyze this in a further study.

In the analysis of the proteinogenic amino acid, longissimus muscle had a greater concentration of methionine in the high‐nutrition group compared to the low‐nutrition group. Martin et al. (2019) reported that, by muscle metabolome analysis, maternal restricted‐feeding and overfeeding during gestation resulted in distinct amino acid metabolite profiles in the longissimus muscle of the offspring in sheep. The longissimus muscle of fetus at late gestation (day 135) contained lower‐ and higher‐abundance of methionine in groups of maternal 60% and 140% nutrition, respectively. This phenomenon was consistent with our result. On the other hand, in the analysis of free amino acid, Glutamic acid concentration indicated greater in the high nutrition compared to the low nutrition group. This would mean that the higher activity of chemical transformations far beyond protein synthesis in the muscle of the high nutrition group compared to the low nutrition group (Walker & van der Donk, 2016). However, further research will be needed to determine how the change of profile of proteogenic amino acids and free amino acids of longissimus muscle by growth and fattening affects metabolism and function of the muscle and the meat quality in mature cattle.

In conclusion, it was suggested that the nutritional management of Wagyu cows during the whole of gestation programed fetal development and performance, including the development of the muscle, fat, bone, and organs. In the present study, we compared the effects of feeding 60% and 120% of the JFSBC requirements for Wagyu dams throughout gestation. The masses of the fetal muscles, bones, and organs were 1.2–1.4 times higher in the high‐nutrition group than in the low‐nutrition group, and the adipose tissue mass was 1.4–3.0 times higher. In particular, the subcutaneous and intermuscular fat depots were larger. It is likely that the excess nutrients tend to predominantly accumulate in the subcutaneous fat and intermuscular fat depots of Wagyu fetuses. Maternal undernutrition is associated with low birth weight, and lighter muscles and fat depots, but also a reduction in size of some important internal organs. Conversely, maternal overnutrition during the whole of gestation does not cause identical balanced (allometric) development of muscle, fat, bone, and organs, which is accompanied by more substantial fat accumulation. The development of organs such as the brain, liver, and pancreas is vulnerable to nutrient deficiency or excess. The present data suggest that the manipulation of maternal nutrition during the entirety of gestation would have significant effects on muscle, adipose tissue, and organ development in the fetuses of Wagyu cattle, and thereby have impacts on the quantity and quality of the meat they subsequently produce.

## CONFLICT OF INTEREST

The authors declare no conflict of interests for this article.
